# Effectiveness of Percutaneous Flexor Tenotomies for the Prevention and Management of Toe-Related Diabetic Foot Ulcers: A Systematic Review

**DOI:** 10.3390/jcm12082835

**Published:** 2023-04-12

**Authors:** María M. Calvo-Wright, Mateo López-Moral, Yolanda García-Álvarez, Marta García-Madrid, Francisco J. Álvaro-Afonso, José Luis Lázaro-Martínez

**Affiliations:** Diabetic Foot Unit, Clínica Universitaria de Podología, Facultad de Enfermería, Fisioterapia and Podología, Universidad Complutense de Madrid, Instituto de Investigación Sanitaria del Hospital Clínico San Carlos (IdISSC), 28040 Madrid, Spain

**Keywords:** diabetic foot, diabetic foot ulcer, digital deformity, flexor tenotomy

## Abstract

There is a high prevalence of digital deformities in diabetic patients, particularly claw toe, which can result in ulceration, often located at the tip of the toe. These lesions are challenging to off-load with conventional devices and frequently lead to infection and high amputation rates. Recent guidelines recommend considering flexor tenotomies to manage these ulcerations and prevent complications. This review, which analyzed 11 studies, aimed to assess the effect of flexor tenotomies on the healing and prevention of diabetic foot ulcers (DFUs) at the toe tip. Satisfactory results were found, with a healing rate of 92% to 100% and a mean healing time of 2–4 weeks. Few mild complications were observed, and the recurrence rate was very low. Transfer lesions were the most prevalent, but simultaneous tenotomy of all toes can eliminate this risk. Flexor tenotomies are a simple, effective, and safe procedure for the treatment and management of DFUs located at the apex of the toes and should be considered part of the standard of care for diabetic feet.

## 1. Introduction

One of the most common complications of diabetes is diabetic foot ulcers (DFUs), which have a lifetime incidence of approximately 19% to 34% [1].

Although the development of diabetic foot ulcers is multifactorial, it is most frequently associated with peripheral neuropathy and foot deformity [2]. Digital deformities such as hammer, mallet, or claw toes are commonly associated with diabetic foot ulceration, with the plantar and dorsal aspects of the toe being the most frequently affected locations [3]. Ulcers on the toes account for 43% to 55.5% of all foot ulcer cases, and while these ulcers are smaller and typically heal faster than the metatarsal head, midfoot, or rearfoot ulcers, they are often underestimated and tend to have higher rates of limb amputations compared to other foot locations [4].

This condition leads to atrophy of the intrinsic foot muscles, specifically the interossei and lumbricals. When intrinsic muscles become dysfunctional and overpowered by the extrinsic muscles (flexor digitorum longus and extensor digitorum longus), the stabilizing action is lost, which can eventually result in claw or hammer toes due to an imbalance between the intrinsic and extrinsic muscles across the metatarsophalangeal joints (MTPJs) and interphalangeal joints (IPJs) [5,6].

A claw deformity is caused by hyperextension of the MTPJ with plantar flexion of the PIPJ and DIPJ. A hammertoe is characterized by hyperextension of the MTPJ and plantar flexion of the PIFJ, but there is no contracture of the DIPJ. In contrast, a mallet toe occurs when the plantar flexion deformity is only found in the DIPJ [7,8].

In those with diabetic neuropathy, toe deformities can increase plantar pressures during midstance and toe-off, leading to the formation of calluses, minor lesions, and, ultimately, toe ulceration, particularly at the tip of the toes [9].

Off-loading and debridement are the basis of treatments to promote healing and prevent the recurrence of tip-toe ulcers [10]. Orthotic interventions such as footwear, toe silicone orthosis, or padding are standard treatments. However, conservative treatment remains unclear, has weak evidence, and often results in poor patient adherence [8,10].

Surgical interventions such as flexor tenotomies (FTs) are often considered when a toe deformity is a risk factor for developing a toe ulcer and when conservative non-operative treatment has been unsuccessful [11]. The International Working Group on the Diabetic Foot (IWGDF) recommends performing digital flexor tendon tenotomies in individuals with diabetes and abundant callus or an ulcer on the apex or distal part of a non-rigid hammer toe to prevent the first ulcer or the development of a recurrent foot ulcer [12]. The procedure consists of locating the flexor tendon by placing it under tension followed by a subsequent transversal incision in the flexor digitorium longus and brevis [11].

Two previous systematic reviews [13,14] have evaluated the effects of flexor tenotomy on the healing and prevention of diabetes-related toe ulcers. To assess the current literature, this review has been conducted due to the recent publication of new studies. Additionally, the effect of flexor tenotomies on the prognosis of further complications, such as toe deformities and transfer lesions, has not yet been evaluated.

The primary aim of this review was to assess the effectiveness of flexor tenotomies in healing and preventing diabetic foot ulcers located on the apex of the toe. The secondary objective was to evaluate the safety and efficacy of flexor tenotomies in preventing and healing diabetic foot ulcers associated with digital deformities.

## 2. Materials and Methods

This systematic review was carried out according to the Preferred Reporting Items for Systematic Reviews and Meta-Analyses (PRISMA) guidelines [15] and has been registered in PROSPERO (a prospective international register of systematic reviews; identification code CRD42023396635).

### 2.1. Literature Search

Three electronic databases were independently searched by two reviewers (MM.C.W and M.L.M) for relevant studies on flexor tenotomies and the healing and prevention of diabetic foot ulcers located on the tip of the toe from inception up to 10 September 2022. The words “flexor tenotomy”, “healing”, “prevention”, and “diabetic foot ulcers” were used as search terms. These keywords were directly combined using the Boolean operator “AND” forming the following search strategies: flexor tenotomy AND healing AND diabetic foot ulcers, flexor tenotomy AND prevention AND diabetic foot ulcers and flexor tenotomies AND diabetic foot ulcers.

### 2.2. Selection Requirements

#### 2.2.1. Inclusion Criteria

Inclusion criteria included (a) studies published in the last 12 years; (b) studies published in English or Spanish; (c) patients with digital deformities associated with diabetes that had either developed a toe ulcer or were at risk of developing a toe ulcer; and (d) studies using a prospective/retrospective case series or case–control design, cross-sectional, or cohort design and randomized clinical trials.

#### 2.2.2. Exclusion Criteria

Exclusion criteria included (a) studies published over 12 years ago; (b) animal trials; (c) articles concerning other types of tenotomies than flexor tenotomies; and (d) articles unrelated to the treatment and prevention of diabetic foot ulcers.

### 2.3. Literature Screening and Data Extraction

Following the deduplication of search results, potential articles were reviewed based on title and abstract. Articles were independently screened by two authors (MM.C.W and M.L.M), and the results were compared. A third reviewer (JL.L.M) resolved any disparity between the authors.

According to the research questions, the general information of each article was arranged in a data chart, including first author, year, study design, objectives, sample, lesion characteristics, type of intervention, and follow-up.

Healing rate and healing time were included in a second table as outcomes, and complications arising from the surgical procedure and adverse effects were included in the second chart.

### 2.4. Quality Evaluation of Included Studies (STROBE Guidelines)

Three independent researchers analyzed the data collected from all articles. As most of the included articles were prospective and retrospective cohort studies (with only one randomized trial included), the quality evaluation was based on the standard STROBE guidelines to ensure a high-quality presentation of observational studies [16]. Raters assessed the adequacy of reported items using the STROBE guideline checklist, which provides a framework for completeness and transparency. The STROBE guidelines checklist has 22 items, including items 1 (title and abstract), 2 and 3 (introduction), 4–12 (methods), 13–17 (results), 18–21 (discussion), and 22 (funding and sponsorship). Two raters (MM.C.W and M.L.M) independently assessed each study using the STROBE guidelines, and a third rater (J.L.L.M.) was involved in achieving a consensus in case of disagreement.

### 2.5. Statistical Analyses

Since the included studies have great heterogeneity in research design, survey time, and outcome indicators, it would be difficult to conduct quantitative analysis, so only qualitative analyses were conducted.

## 3. Results

### 3.1. Literature Retrieval

**Figure 1:**
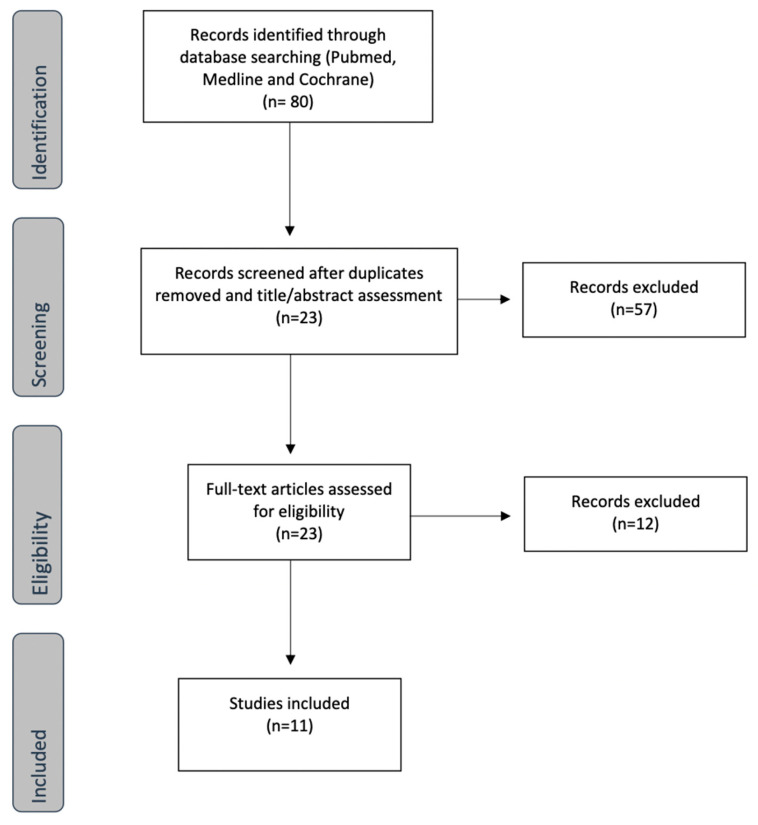
Flowchart of identified studies.

In the first search applying the inclusion criteria, 80 articles were identified. After eliminating duplicates and reading the title and abstract, 23 articles were selected for full-text evaluation. Ultimately, 11 studies were included for analysis. Figure 1 shows the literature screening process.

### 3.2. Characteristics of the Included Studies

Among the included literature, ten studies were case series studies, of which seven were retrospective [17,18,19,20,21,22,23] and three prospective [24,25,26]. One randomized clinical trial was also assessed [27]. The eleven studies included 770 flexor tenotomies performed in diabetic patients.

In the study by Schmitz et al. [22], 101 tenotomies to treat digital lesions in diabetic and non-diabetic patients were evaluated; those with a curative indication in 64 diabetic feet could be evaluated in isolation, but the prophylactic group with 13 diabetic feet and 4 non-diabetic feet were analyzed together. Scheepers et al. [17] and Tamir et al. [21] also included a minority of neuropathic patients without DM in their studies but did not specify the number of tenotomies performed in diabetic patients; therefore, they could not be assessed independently.

Among the total of 770 tenotomies, 387 had a curative indication, and 388 were prophylactic; six studies included both indications, two evaluated only prophylactic tenotomies, and three evaluated only the curative indication. The study by Hedegaard Andersen et al. [23] evaluating both indications showed that in the curative tenotomy group, 14 interventions were also considered prophylactic because the patient had another finger with a preulcerative lesion (PUL) in addition to the ulcerated toe.

The studies included patients who had undergone FT to treat one or more lesions located in the apex of the toes associated with a flexible or semi-flexible digital deformity, except for the RCT by Andersen et al. [27] and the study by Smith et al. [24], in which participants with rigid digital deformities were not excluded.

Tamir et al. [21] evaluated flexor tenotomies for the treatment of DFUs in other locations than the tip and combined this technique with extensor tenotomies in selected participants in addition to performing isolated extensor tenotomies depending on the location of the lesion; these cases were not included in the outcome analysis of the present systematic review.

Another study [23] included ulcers and preulcerative lesions at locations associated with digital claw, hammer, or mallet deformities that differed from the tip of the toe, and the results for all lesion types were evaluated together.

The etiology of the lesions was neuropathic in most cases, although some articles included neuroischemic lesions [17,18,20,21,22,23,24]. The presence of soft tissue infection was an exclusion criterion common to all studies, but several articles included lesions with osteomyelitis (OM) [19,21,24].

Ulcer evolution times ranged from 1 to 9 months, although, for most of the studies, the average preintervention wound evolution time was around 3 months.

Regarding the surgical procedure technique, there were studies in which only the flexor digitorum longus was sectioned [17,21,22] and others in which the flexor digitorum longus and flexor digitorum brevis were approached together [17,19,20,26,27], with the incision placed proximally or distally depending on the approach. The tenotomy was performed with a scalpel [17,18,19,20,21,22,24,26]; in some cases, a percutaneous needle was used [23,24,25,27].

Post-surgical follow-up time ranged from 6 [19,24] to 28 months [18]; five studies followed patients for around 1 year [17,22,25,26,27], and three articles followed patients for approximately 2 years [19,21,23]. The research characteristics are shown in Table 1.

### 3.3. Quality of the Reporting

Items 9 (bias), 10 (study size), 19 (limitations), and 21 (generalizability) were the most poorly completed by the included studies. Table 2 shows the overall rating for the STROBE checklist.

### 3.4. Screened Outcomes

The results obtained concerning the healing rate and healing time, complications arising from the surgical procedure, and adverse effects are shown in Table 3.

#### 3.4.1. Healing Rates and Mean Healing Times

Data on healing rates and healing times were satisfactory for all studies, with healing rates ranging from 92% to 100% and healing times around 2–4 weeks, except for the article by Kearney et al. [18], which showed a mean healing time of 5–7 weeks. The shortest healing time was observed in the cohort of Smith et al. [24], considering that most wounds were superficial and free of infection. Studies agree that lesions with infection and deeper tissue penetration had longer healing times [19,21].

#### 3.4.2. Ulceration and Recurrence Rates

The articles that evaluated tenotomies with prophylactic indication reflected rates of progression to active ulcer and recurrence rates of 0%, except for Hedegaard Andersen et al. [23] and Schmitz et al. [22], who showed in their studies that preulcerous lesions treated with TF progressed to ulceration, but in a very low percentage.

In the study by Schmitz et al. [22], this event was observed in two patients, but they did not specify the location or whether the patient was diabetic; assessing two simultaneous populations is a limitation in this respect. The follow-up period in the study by Hedegaard Andersen et al. [23] was longer than in other studies. Additionally, in other studies, the intervention of each toe was assessed as one procedure, whereas in this case, one procedure could include one to ten toes; if the ulceration rate per toe and per procedure is calculated, the ulceration rate is 3%.

#### 3.4.3. Complications Arising from the Surgical Procedure

Regarding complications, six articles [18,21,22,23,24,27] reported on post-surgical events such as pain and hematoma associated with the operation or infection, which were not considered serious.

Therefore, the studies agree that tenotomies are simple and safe procedures that effectively unload the apex of the toes by reducing digital deformity. Mens et al. [24] used objective biomechanical and musculoskeletal tests to demonstrate this off-loading effect; their findings show a large off-loading effect with a >50% reduction in pressure on the tip toe in line with the hypothesized causal mechanism of this minimally invasive surgery in the prevention of toe ulcers.

#### 3.4.4. Adverse Events

Complications observed during follow-up were mostly transfer injuries and reulcerations. Several articles [19,22,27] treated transfer injuries in another episode of intervention using flexor tenotomies and showed satisfactory results, and in some cases, additional osteotomies were necessary [20].

The studies [18,19,20,21,22,23] had a total of 14 lesions that did not heal during follow-up, and in two studies, reinterventions had to be performed due to insufficiency of the initial procedure. A total of nine amputations were also found in the studies, three of which were associated with the ulcer treated with tenotomy; these lesions had osteomyelitis. Kearney et al. [18] associated the non-healing case with the presence of a pre-existing hallux amputation; in the article by Van Netten et al. [19], the ulcers that did not heal had an infection and penetrated the bone, but most of the ulcers with these characteristics did heal, almost half of them without complications.

## 4. Discussion

The evaluated literature presents favorable and satisfactory data regarding the effectiveness, efficacy, and safety of flexor tenotomies in treating and preventing DFUs located on the tip of the toes, which is consistent with the results obtained in previous reviews. This review quantitatively analyses outcomes, using healing rate and mean healing time to determine the effectiveness of flexor tenotomies; this reflects a strength of the study.

In addition, this review reported on the most prevalent complications resulting from flexor tenotomies, which is the main strength of the present study because these effects have not been evaluated before in the literature. Transfer injuries were the most common adverse effect observed. It should be noted that after flexor tenotomy, the adjacent toe (due to structural and functional changes) may develop a transfer injury due to increased pressure, which can be considered serious because it may result in ulceration, infection, and subsequent amputation.

Regarding these complications, Lopez-Moral et al. conducted a study evaluating the long-term clinical outcomes of patients who underwent isolated versus multiple flexor tenotomies [26]. They found a higher rate of reulceration due to transfer injuries in the isolated tenotomy group, a higher prevalence of hyperkeratosis and deformities in adjacent toes, and higher peak barefoot pressure and pressure/time integral in toes without tenotomy in the isolated tenotomy group. These results support the idea that patients with a history of ulceration or incipient callus on the tip of the toes should undergo percutaneous flexor tenotomies on all toes to reduce long-term complications. Consistent with these findings, Hedegaard Andersen J et al. observed that the risk of transfer injury was eliminated in patients who underwent TF of all toes simultaneously [23].

In terms of limitations, most of the articles evaluated do not include a significant sample of patients with neuroischemic ulcers. In the study performed by Scheppers T et al., which included a patient with PAD, it was found that this condition was not associated with complications or delayed healing, likely due to the minimally invasive nature of the procedure [17]. The authors also reported that osteomyelitis did not affect healing but that patients took longer to heal. This finding is consistent with existing data and general principles regarding diabetes-related foot ulcers and the delay in postoperative healing caused by osteomyelitis.

Furthermore, the studies evaluated are mainly retrospective and lack high-quality evidence for analysis. There is only one RCT in the literature that compares tenotomies with SOC, highlighting the need for more of this type of study. Future research should include quantitative data analysis to enable meta-analysis, but this requires more RCTs comparing two interventions.

Regarding digital deformities, it is true that the articles define them differently, and in most cases, a complete evaluation of them is not performed, which may lead to erroneous indications for these techniques or associated complications. Moreover, there is no consensus regarding the technique and the influence of sectioning one or both flexors. Scheppers T et al. reported iatrogenesis with the section of the plantar plate resulting in a hyperextended toe that required amputation [17]. Van Netten et al. observed a patient in whom both flexors were severed, resulting in dorsiflexion of the AMTF that developed ulceration [19].

To avoid these complications, an assessment of dynamic deformities during gait should be included as a pre-surgical evaluation. Additionally, to maximize the probability of successful surgical outcomes, each patient’s biomechanics should be assessed in a loading situation, and the etiology of the toe deformity should be analyzed [7,8].

The systematic use of pressure-relieving therapy with therapeutic footwear, close follow-up, correct antibiotic prophylaxis, and control of comorbidities (multifactorial approach) are essential for successful therapy [26,27], and studies that apply these principles have shown better results. Rasmussen et al. did not follow up with patients monthly after healing, as recommended by the IWGDF guidelines; therefore, the finding of reulceration events over a longer time than that identified other studies could be related to this [20].

Several studies report the use of plantar orthoses and appropriate footwear after surgery, with some studies highlighting their benefits [20,25,26]. However, other articles indicate that patients could do without custom-made or special footwear after surgery [17].

## 5. Conclusions

Flexor tenotomies are an effective treatment for neuropathic UPDs located at the distal end of the toes, showing a high healing rate with a short healing time. They are also an excellent prophylactic procedure, demonstrating low rates of ulceration and recurrence and being effective in preventing UPD in the presence of digital deformity or preulcerative signs, provided their indication is correct. Therefore, these techniques should be included in the day-to-day standard of care for diabetic feet.

The presence of mild ischemia or osteomyelitis should not be considered a contraindication for the practice of these procedures. However, in these cases, there are longer healing times and a higher risk of complications during follow-up. Transfer injuries are the most prevalent secondary complication; performing a tenotomy of all toes simultaneously eliminates this risk and other complications. Therefore, it is advisable to perform multiple tenotomies rather than isolated ones. Further RCTs are required to support these conclusions with more evidence, and future research needs to include ischemia and infection data.

## Figures and Tables

**Table 1:** General characteristics of the included studies.

First Author Year	Study Design	Objectives	Sample	Lesion Characteristics	Surgical Intervention	Follow-Up
Schepers T. 2010, [17]	retrospective	To assess the results of using flexor tenotomies to treat ulcers in flexible claw toes.	23 patients - 15 with diabetes - 5 DM + PAD 25 ulcers 17 PULs	- Wagner 0–2 (95%) - Location: the tip of the toes - The mean time of evolution = 6.8 months - Deformity: flexible claw toe	- Technique: FDL and FDB sectioned - Total *n* = 42 - Curative *n* = 42 - Prophylactic NA	11 months
Kearney TP. 2010, [18]	retrospective	To evaluate the effectiveness and safety of percutaneous tenotomy of the flexor digitorum longus for healing neuropathic ulcers in the tip of the toes.	48 patients with diabetes - 21 PAD 58 ulcers	- Location: the tip of the toes - Deformity: flexible	- Technique: FDL sectioned - Total *n* = 58 - Curative: *n* = 58 - Prophylactic NA	28 months
Van Netten JJ. 2013, [19]	retrospective	To report healing rates and healing times and to investigate the influence of preoperative treatment, time of ulcer evolution before tenotomy, and location or presence of infection on healing and healing time. They also wanted to describe the advantages of using this technique as a prophylactic intervention in diabetic patients with claw or hammertoes.	33 patients with diabetes - 31 DN - No PAD 38 ulcers	- Texas 3b majority - Location: tip of toes - Mean time of evolution = 96 days - Deformity: flexible hammer or claw toe - OM included	- Technique: FDL and FDB sectioned - Total: *n* = 47 - Curative *n* = 38 - Prophylactic *n* = 9 * 8 transfer tenotomies because they were performed on the same foot after an initial procedure	23 ± 11 months
Rasmussen A. 2013, [20]	retrospective	To examine the effectiveness of a modified flexor tenotomy technique to prevent and heal neuropathic and neuroischemic ulcers located on the tip of the toe in the presence of claw or hammertoe deformity in diabetic patients.	38 patients - 16 with 27 ulcers - 22 with 38 PULs	- Neuroischemic ulcers - Location: tip of toes - Mean time of evolution = 15 weeks - Deformity: flexible hammer or claw toe	- Technique: FDL and FDB sectioned - Total: *n* = 65 - Curative *n* = 27 - Prophylactic *n* = 38	6 months
Tamir E. 2014, [21]	retrospective	To report on the performance of percutaneous flexor and extensor tenotomies for treating neuropathic ulcers.	55 patients with diabetes * Patients with critical ischemia were excluded	- They affected mostly skin and subcutaneous cellular tissue - Location: tip, dorsum, interdigital and metatarsal head - Mean time of evolution = 33 weeks - Cellulite excluded - OM included	- Technique: FDL sectioned - Total: *n* = 103 - Curative *n* = 103 - Prophylactic NA	22 months
Schmitz P. 2019, [22]	retrospective	To assess whether percutaneous flexor tenotomy is an effective intervention to treat and prevent toe ulcers and whether prophylactic percutaneous tenotomy is a safe and effective way to prevent ulceration.	101 feet included 77 with DFS - 64 DFUs - 13 PULs	- 64 with DN - 1 with PAS - 18 DN + PAS - Deformity: flexible claw toe - Mean time of evolution = 124 days	- Technique: FDL sectioned - Total in DFS group: *n* = 77 - Curative *n* = 64 - Prophylactic *n* = 13 - * In both groups = curative 84 and prophylactic 17	13.4 months
Hedegaard Andersen J. 2019, [23]	retrospective	To show the outcome of percutaneous needle tenotomies and the benefit of flexor tenotomies as a treatment for claw, hammer, and mallet toes in people with diabetes.	81 patients with diabetes - >Type II - DN - 20% PAS	- Neuropathic, ischemic, and neuroischemic - Location: tip, dorsum, interdigital, and metatarsal head - Mean time of evolution = 4.5 weeks - Deformity: claw, hammer, or mallet	- Technique: Percutaneous needle - Total: *n* = 106 - Curative *n* = 36 - * (14 were considered curative + prophylactic) - Prophylactic: *n* = 70	97 weeks
Smith SE. 2020, [24]	prospective	To show the effectiveness and usefulness of percutaneous flexor tenotomies for the healing of neuropathic ulcers at the distal end of the toes performed in an outpatient setting and to show the effectiveness of percutaneous flexor tenotomies for the prevention of progression of preulcerative toe lesions to diabetic foot ulcers.	23 patients with diabetes - without PAS 11 ulcers 41 PULs	- Texas 1A majority - Location: tip of 2° and 3° toe (majority) - Mean time of evolution = 105 days - Deformity: >flexible claw toe	- Technique: FDL or FDL and FDB sectioned with needle or scalpel - Total: *n* = 76 - 51 FDL and 25 FDL + FDB - Curative *n* = 11 - Prophylactic *n* = 65	6 months
Mens MA. 2022, [25]	prospective	To evaluate the effect of percutaneous flexor tenotomy in diabetic patients on plantar pressure, toe angulation, and ulcer recurrence.	14 patients with diabetes - ½ with PAS 19 feet 50 toes	- PUL and history of ulcer on the apex of the toes - Deformity: flexible or semi-flexible	- Technique: percutaneous needle - Total: *n* = 19 - Curative NA - Prophylactic: *n* = 19	14.4 months
López-Moral M. 2022, [26]	prospective	To evaluate the long-term clinical outcomes of patients who underwent isolated percutaneous flexor tenotomies versus multiple tenotomies to treat previous toe deformities and diabetic foot ulcers.	23 patients with diabetes - DN - without critical ischemia 31 feet	- PUL and history of ulcer on the apex of the toes - Deformity: flexible	- Technique: FDL and FDB sectioned with percutaneous needle - Total: *n* = 99 - Curative NA - Prophylactic *n* = 99 * 31 feet operated 11 with isolated tenotomies - 20 with several tenotomies	1 year
Andersen J. 2022, [27]	RCT	To examine the ability of tenotomies to prevent and treat hammertoe-associated ulcers in diabetic patients.	96 patients with diabetes 16 ulcers 79 PULs	- Lesions associated with flexible, semi-flexible, or rigid hammer toe deformity	- Technique: FDL sectioned - Total: *n* = 47 - Curative *n* = 8 - prophylactic *n* = 39 4 subgroups: PUL with SOC PUL with tenotomies + SOC DFU with SOC DFU with tenotomies + SOC	1 year

DM, diabetes mellitus; PAD, peripheral arterial disease; DN, diabetic neuropathy; PUL, preulcerative lesion; FDL, flexor digitorum longus; FDB, flexor digitorum brevis; NA, not applicable; OM, osteomyelitis; DFS, diabetic foot syndrome; DFU, diabetic foot ulcer; RCT, randomized controlled trial; SOC, the standard of care. *, additional information.

**Table 2:** The overall rating for Strengthening the Reporting of Observational Studies in Epidemiology (STROBE).

Item Number–STROBE Guidelines																							
	1(a)	1(b)	2	3	4	5	6	7	8	9	10	11	12	13	14	15	16	17	18	19	20	21	22
Schepers T. 2010, [17]	No	Yes	Yes	No	Yes	Yes	No	Yes	Yes	No	No	No	No	Yes	Yes	Yes	Yes	No	Yes	No	Yes	No	No
Kearney TP. 2010, [18]	Yes	Yes	Yes	Yes	Yes	Yes	Yes	Yes	No	No	No	No	No	Yes	Yes	Yes	Yes	Yes	Yes	Yes	Yes	Yes	Yes
Van Netten JJ. 2013, [19]	Yes	Yes	Yes	Yes	Yes	Yes	Yes	Yes	Yes	No	Yes	No	Yes	Yes	Yes	Yes	Yes	No	Yes	No	Yes	Yes	Yes
Rasmussen A. 2013, [20]	Yes	Yes	Yes	Yes	Yes	Yes	Yes	Yes	Yes	No	No	Yes	Yes	Yes	Yes	Yes	Yes	Yes	Yes	No	Yes	No	No
Tamir E. 2014, [21]	Yes	Yes	No	Yes	Yes	No	Yes	No	No	No	No	No	Yes	Yes	Yes	Yes	Yes	Yes	Yes	No	Yes	No	Yes
Schmitz P. 2019, [22]	Yes	Yes	Yes	Yes	Yes	Yes	No	Yes	No	No	Yes	Yes	No	Yes	Yes	Yes	Yes	Yes	Yes	Yes	Yes	No	Yes
Hedegaard Andersen J. 2019, [23]	Yes	Yes	Yes	Yes	Yes	Yes	No	Yes	Yes	No	No	No	No	Yes	Yes	Yes	Yes	Yes	Yes	Yes	Yes	No	Yes
Smith SE. 2020, [24]	Yes	Yes	Yes	Yes	Yes	Yes	Yes	Yes	Yes	Yes	No	Yes	Yes	Yes	Yes	Yes	Yes	Yes	Yes	No	Yes	No	Yes
Mens MA. 2022, [25]	No	Yes	Yes	Yes	Yes	No	Yes	Yes	No	No	No	Yes	Yes	Yes	Yes	Yes	Yes	Yes	Yes	Yes	Yes	Yes	No
López-Moral M. 2022, [26]	Yes	Yes	Yes	Yes	Yes	Yes	Yes	Yes	Yes	Yes	Yes	No	Yes	Yes	Yes	Yes	Yes	No	Yes	No	Yes	Yes	Yes
Andersen J. 2022, [27]	Yes	Yes	Yes	Yes	Yes	Yes	Yes	Yes	Yes	Yes	Yes	Yes	Yes	Yes	Yes	Yes	Yes	Yes	Yes	Yes	Yes	Yes	Yes

**Table 3:** Screened outcomes.

Researchers	Healing Rate (%)	Mean Healing Time	Adverse Events	Surgical Complications
Schepers T et al. (2010), [17]	100%	3.6 weeks	- 1 recurrence - 1 minor amputation	- Section of plantar plate
Kearney TP et al. (2010), [18]	98.3%	40–52 days	- Reulceration rate in the same site 12.1% (mean time of appearance 13.9–15.2 months) - Post-surgical infection rate 5.2% not in place of incision - 1 unhealed lesion	- No complications
Van Netten JJ et al. (2013), [19]	92%	22 ± 26 days	- 3 minor amputations (of non-healing ulcers) - 7 reulcerations - 1 dorsiflexed metatarsophalangeal joint	- No complications
Rasmussen A et al. (2013), [20]	93%	21 days	- 3 recurrences - (One healed after repeating the tenotomy) - 2 transfer lesions - 2 unhealed ulcers	- 1 insufficient procedure
Tamir E et al. (2014), [21]	98%	4 weeks	- 2 unhealed ulcers - 9 transfer ulcers - 3 ruptures of the skin secondary to toe extension	- 1 mild infection - 1 patient with plantar pain
Schmitz P et al. (2019), [22]	93.8%	22 days	- Curative group: 4 infections, 1 minor amputation, 8 recurrences, and 2 transfer ulcers and 4 unhealed ulcers - Prophylactic group: 2 ulcerations	- 1 bleeding, 1 reintervention
Hedegaard Andersen J et al. (2019), [23]	94%	28 days	Curative group: - 5 recurrences - 2 unhealed Prophylactic group: - 6 progressions to active ulcer - 4 extensor tenotomies 25 transfer lesions (7 ulcers and 18 PULs) 4 amputations (3 minor and 1 major)	- 4 insufficient procedures that were repeated - Plantar pain (14%)
Smith SE et al. (2020), [24]	100%	10.2 ± 4.3 days	- Transfer lesions (15.5%) - 3 ulcers and 3 PULs	- Post-surgical infection (2.8%)
Mens MA et al. (2022), [25]	NA Recurrence 0%	NA	- No adverse events	- Without complications
López-Moral M et al. (2022), [26]	NA Recurrence 0%	NA	Insolated tenotomies: - 8 transfer lesions in 9 weeks (72.7%) - 11 adjacent HK increased + claw toes in 5 and a half weeks (100%) - 9 minor lesions in 6 and a half weeks (81%) Multiple tenotomies: - 16 floating toes (80%)	- Without complications
Andersen J et al. (2022), [27]	100% Recurrence 0%	Days (7–26)	- Curative group: no adverse effects - Prophylactic group: 5 transfer lesions, 2 PULs, and 3 ulcers	- Curative group: 2 with pain and 2 with hematomas - Prophylactic group: 21 with pain 7 with hematomas, and 1 patient with a feeling of loss of balance

%, percentage; PUL, preulcerative lesion; NA, not applicable; HK, hyperkeratosis.

## Data Availability

The data are available previous request to corresponding author.
